# Patterns of agreement between children and parents in SDQ subscales: latent class and latent profile analyses

**DOI:** 10.1186/s13034-026-01206-1

**Published:** 2026-11-11

**Authors:** Ferdinand Keller, Alexandra N. Langmeyer, Jost Reinecke, Robert Schlack

**Affiliations:** 1 Department of Child and Adolescent Psychiatry, Psychosomatics and Psychotherapy, University Hospital Ulm, Ulm, Germany; 2 Department of Children and Childcare, German Youth Institute, Munich, Germany; 3 Faculty of Sociology, Bielefeld University, Bielefeld, Germany; 4 Department of Epidemiology and Health Monitoring, Robert Koch-Institute, Unit of Mental Health, Berlin, Germany

**Keywords:** Adolescent mental health, Informant discrepancy, SDQ, KiGGS, Latent class analysis, Latent profile analysis, Configural frequency analysis

## Abstract

**Background:**

Multi-informant assessment is considered best practice in child and adolescent mental health research, yet agreement between child and parent reports is often only moderate. The present study examined patterns of agreement and discrepancy between children’s and parents’ ratings on the Strengths and Difficulties Questionnaire (SDQ) using both variable-centered and person-centered approaches in a nationally representative German sample.

**Method:**

Data are used from Wave 2 of the German Health Interview and Examination Survey for Children and Adolescents (KiGGS), including 5,224 child–parent dyads of adolescents aged 11–17 years. To identify latent patterns of agreement and discrepancy, latent profile analyses (LPA) and latent class analyses (LCA) were conducted. Configural frequency analysis (CFA) was applied to test for patterns across the SDQ domains.

**Results:**

Descriptive analyses showed moderate agreement across SDQ subscales, with children generally reporting higher levels of difficulties than their parents, particularly for the subscales Emotional Problems, Peer Problems, and Hyperactivity/Inattention. Agreement varied substantially across items, with higher concordance for more observable behaviors and lower agreement for internal or evaluative symptoms. The scale-level LPA identified six profiles differing in symptom severity and degree of child–parent agreement. Two profiles were characterized by systematic child-greater-than-parent discrepancies, whereas no clear parent-greater-than-child profile emerged. Item-level LCAs across all five SDQ subscales revealed both concordant and discordant classes, including distinct patterns in which either children or parents consistently reported more symptoms. Across domains, concordant low-symptom classes represented the largest groups, while concordant high-symptom classes were smaller but clearly identifiable. Cross-domain analyses with CFA showed only limited evidence for a generalized discrepancy type across all SDQ domains. However, a second-order LCA identified small but meaningful subgroups characterized by systematic child-greater-than-parent or parent-greater-than-child reporting patterns across multiple domains.

**Conclusions:**

Although the results obtained using different analytical approaches did not fully converge and need to be replicated, the findings clearly suggest that the discrepancies between informants are structured and domain-specific, and cannot simply be explained by measurement error. Person-centered approaches provide important additional insights beyond conventional correlational analyses and may improve the interpretation of multi-informant SDQ assessments in both research and clinical contexts.

**Supplementary Information:**

The online version contains supplementary material available at https://doi.org/10.1186/s13034-026-01206-1.

## Introduction

Understanding the mental health of children and adolescents is a critical area of research, given its implications for individual development and societal well-being [1]. In this context, multi-informant assessments have become a best-practice approach for evaluating children’s mental health problems [2–4]. Complementary to this, a multi-method approach combining clinical interviews, rating scales, and observational data is recommended to increase diagnostic accuracy [5, 6].

Among available instruments, the Strengths and Difficulties Questionnaire (SDQ; [7]) is one of the most widely used tools for both clinical and population-based applications. Its brevity, validated translations, and parallel versions for children, parents, and teachers make it particularly suitable for large-scale studies [8–10]. The SDQ comprises 25 items evenly distributed across five subscales—Emotional Problems, Peer Problems, Hyperactivity/Inattention, Conduct Problems, and Prosocial Behavior—with the first four forming the Total Difficulties Score (TDS). Psychometric properties and measurement invariance of the SDQ have been examined in several studies, with findings generally supporting measurement invariance across subgroups [11–14], informants [15] and longitudinally [16, 17].

### Empirical evidence on child–parent agreement in the SDQ

A substantial body of research has examined the level of agreement between child and parent reports on the SDQ. Overall, findings suggest that both perspectives are valuable and suitable for diagnostic purposes [18, 19]. However, several studies also indicate notable discrepancies between informants, e.g. [2, 8, 20]. Cross-informant data have been collected in various countries [21–27]. German studies include both smaller clinical samples [28] and large representative surveys such as the pairfam study [29] and the KiGGS study [10], which provides the data foundation for the present analysis.

Across this body of work, findings consistently show low to moderate correlations between children’s and parents’ SDQ scores. A meta-analysis by Bergström and Baviskar [30] reported average correlations of *r* = .30–0.38 for subscales and *r* = .46 for the TDS in general population samples, and *r* = .40–0.53 for subscales and *r* = .38 for the TDS in clinical samples. Some studies have extended these analyses to the item level, for example by applying coefficients for agreement between children and parent for each SDQ-item [23] or IRT approaches for comparing category thresholds [21, 31]. Others have examined agreement at the categorical level, employing the SDQ’s standard three-band classification (“normal,” “borderline,” “abnormal”) and corresponding cutoff scores using cross-tables analyses [9, 25, 26, 32].

### Methodological challenges and recent developments

While categorical classifications are often used for screening, they entail a substantial loss of information and statistical power. Markon et al. [33] concluded from results of two meta-analyses that a 15% increase in reliability and 37% increase in validity can be expected through adoption of a continuous over discrete measure of psychopathology alone. Moreover, the SDQ shows only moderate reliability for some subscales [34], which, combined with rigid cutoff values, complicates precise interpretation near thresholds [35]. These limitations underline the importance of continuous and probabilistic approaches.

Beyond these measurement issues, informant discrepancies can stem from methodological, developmental, and contextual factors. For example, parental psychological distress or parenting stress may bias parent ratings [8, 36], while perceptual awareness of symptoms [18, 37] and individual norms for behavior [28] also contribute to variation. Despite extensive discussion of these factors [6, 38], less is known about how different methodological approaches influence observed agreement levels.

Early studies typically relied on simple correlation coefficients across the full scale range to assess child–parent agreement [30]. More recent research, however, has applied structural equation modeling (SEM) to correct for measurement error and distinguish informant-specific variance, although some authors are skeptical to see informant discrepancies between children and parent reports merely as ‘measurement error’ [6, 38]. Booth et al. [8] used Latent Difference Score models to estimate true-score discrepancies, showing that adolescents reported more difficulties than their parents, moderated by gender and psychological distress. A study by Brocker et al. [39] found moderate agreement between parent and child SDQ reports, operationalized via correlations of summed scores and discrepancy indices based on differences between parent and child ratings. Rogge et al. [15] applied multigroup confirmatory factor analysis, finding configural but only partial scalar invariance between parent and child SDQ versions, indicating that most—but not all—items reflect comparable latent constructs across informants. These findings suggest that discrepancies reflect both true perceptual differences and measurement-related factors.

### Person-centered methods for identifying subgroups of agreement and discrepancies

Person-centered approaches have been introduced to better capture heterogeneity in informant agreement patterns. These mixture model approaches assume that a population is composed of latent subgroups that differ in their patterns of response similarity between informants. Two primary approaches are typically distinguished: latent class analysis (LCA), which models categorical item responses, and latent profile analysis (LPA), which operates on continuous scale scores [40, 41]. Such approaches can reveal distinct subgroups in which parents and children either show high convergence or pronounced discrepancies in their reports. Only a few studies have applied these methods to the SDQ or similar instruments. Makol et al. [37] used LCA to analyze the convergence between adolescents’ and parents’ reports on internalizing problems. After dichotomising their sample into two groups, one with clinical significant problems and one without, their analyses identified four latent classes reflecting distinct reporting constellations: (1) both adolescents and parents reporting few internalizing symptoms, (2) both reporting high levels, (3) adolescents reporting more problems than parents, and (4) parents reporting more problems than adolescents. These groups correspond to the manifest combinations derived from dichotomized “low/high” scores but are modeled probabilistically, taking into account measurement error. The authors further compared these classes in terms of demographic, clinical, and treatment-related variables, demonstrating meaningful external validity of the latent classes. Using a related approach, Vugteveen et al. [19] applied latent profile analysis based on the full range of SDQ subscale scores from both adolescents and parents. They identified six distinct SDQ profiles that differed in both the type and severity of reported difficulties across informants (% in Community sample): (1) no difficulties profile (55%), (2) borderline hyperactivity difficulties profile (15%), (3) borderline conduct and social difficulties profile (17%), (4) emotional difficulties profile (9%), (5) emotional and social difficulties profile (4%), and (6) overall difficulties profile (1%). Importantly, these profiles captured both variation in symptom domains and severity levels, ranging from profiles within the normal range to those characterized by multiple difficulties in the abnormal range. Profiles combining information from both informants predicted mental health service use and DSM-IV diagnoses more accurately than those derived from single-informant data [19]. This finding underscores the added value of jointly modeling parent and child reports. Building on this work, Keller et al. [31] developed a more fine-grained item-level LCA for the SDQ. For each subscale, the five child-rated and five parent-rated items were analyzed jointly (ten items per model) to identify latent classes characterized by specific patterns of agreement or disagreement. The analyses revealed that, for the subscales Emotional Problems and Prosocial Behavior, four distinct latent classes emerged: two classes showing largely homogeneous responses between adolescents and parents, and two classes showing discrepant response patterns across all five item pairs. In one of these discrepant classes, adolescents consistently rated themselves as experiencing fewer difficulties (or higher prosociality) than their parents reported, while in the other class the opposite pattern was observed. These item-level differences translated into substantial discrepancies at the subscale level, with large effect sizes ranging from |d| = 1.44 to 2.54. For the subscale Conduct Problems, in contrast, only two homogeneous classes were identified, suggesting higher agreement in this domain. Keller et al. [31] also found that the correlational structure between adolescents’ and parents’ SDQ responses was moderate but consistent across items and subscales. However, their study was limited by the available data in the German family panel pairfam, which included only three of the five SDQ subscales. Consequently, important dimensions such as Peer Problems and Hyperactivity/Inattention could not be examined.

Taken together, these findings suggest that the modest convergence observed in parent– child agreement is not simply indicative of generally low agreement, but can instead be disentangled into distinct latent subgroups characterized either by high concordance or by patterns of discrepancy between informants [31, 37].

### Aims and research questions of the present study

The KiGGS study, with full SDQ coverage across all five subscales, now enables a comprehensive investigation of child–parent agreement in a large, nationally representative German sample. Building on previous findings, the present study combines correlational, item-level, and person-centered approaches to examine the structure, level, and pattern of agreement between child and parent SDQ reports.

Based on these considerations, the present study addresses the following research questions:

1. How are children’s and parents’ SDQ reports related on the manifest level, both across subscales and individual items, and do these associations correspond to findings reported in previous literature? To answer this research question, descriptive statistics, correlational measures, and different coefficients to assess the degree of agreement are used.
2. ​What patterns of agreement and disagreement between child and parent reports can be identified by latent modeling approaches at the subscale (2a) and item-levels (2b)?
  Latent profile analysis (LPA) is used to test consistency at scale level, and Latent class analysis (LCA) is calculated for each SDQ subscale to test consistency at item-​level.
3. To what extent are the child–parent agreement and disagreement patterns identified by the item-level LCAs for each SDQ subscale consistent across subscales?
  For example: Have child-parent dyads with discrepant patterns in the subscale Emotional Problems the same discrepancies in the subscale Peer Problems? To address this question, we examine whether children classified into particular agreement or disagreement patterns in one SDQ subscale tend to show similar patterns in other subscales. Cross-tabulations of the subscale-specific LCA solutions, configural frequency analysis (CFA), and a second-order LCA are used to examine these cross-subscale patterns.

## Methods

### Sample

Data was taken from the German Health Interview and Examination Survey for Children and Adolescents (KiGGS) which is a cohort-sequential study that collected comprehensive and representative data on the health status of children and adolescents in Germany. The study was part of the German health monitoring system established at the Robert Koch Institute (RKI), on behalf of the German Federal Ministry of Health. The current analyses rely on Wave 2, the second follow-up survey conducted between 2014 and 2017 as a combined health interview and examination survey for children and adolescents aged 0 to 17 years [42, 43]. It includes a psychopathological assessment of children and adolescents from 11 to 17 years using both the parent- and the self-administered SDQ. The sample of completed SDQ questionnaires for the children is *n* = 5771 and for the parents *n* = 5903. Since latent class analyses on the item level are an essential part of our analyses, we decided to include only those pairs where all items had complete data. Thus, the sample for the following analyses includes 5224 child-parent pairs (90.5% of the children, 88.5% of the parent sample) with no missing data on item-level. The parent questionnaires were completed by mothers (75.7%), fathers (6.4%), or jointly by both parents (17.3%), with the remaining questionnaires completed by other caregivers (grandparents or other relatives, foster parents, or legal guardians). Mean age of the children was 13.84 years (SD = 1.97; range: 11 to 17 years) and the proportion of girls was 52% (48% boys). Comparisons of the current sample with the sample of all, i.e., incomplete responses to the SDQ questionnaires of both children and parents by means of the R package mice result in no significant differences regarding the particular SDQ subscales as well as the SDQ total scores. Distributions of children’s sex and age are also not different between the samples of incomplete and complete data.

### Measurements and scales

The Strengths and Difficulties Questionnaire (SDQ; [7]) consists of 25 items reflecting the five dimensions Emotional Symptoms, Peer Problems, Hyperactivity/Inattention, Conduct Problems, and Prosocial Behaviour with the first four forming the Total Difficulties Score (TDS). The subscales consist of five items each. The items are rated on a 3-point Likert scale as “not true” (0), “somewhat true” (1), or “certainly true” (2). Five items of the TDS are reverse scored (cf. Table 1).

The internal consistency (Cronbach’s alpha) of the subscales was calculated as follows (child, parent): Emotional Problems (0.71, 0.68), Peer Problems (0.58, 0.65), Hyperactivity/Inattention (0.68, 0.78), Conduct Problems (0.45, 0.59), Prosocial Behaviour (0.66, 0.66).

### Statistical analyses

For answering research question 1 the agreement between subscale scores was assessed by Pearson and Spearman correlation coefficients. Agreement on the item level was evaluated by tests for contingency tables (chi²-Test, Cramer’s V) and coefficients of agreement. An often-used coefficient for agreement between two observers is Cohen’s Kappa. Kappa, however, is affected by the relative frequencies of the categories (prevalence) and differences in the frequency of occurrence between observers (bias) [44]. Kappa may therefore produce counterintuitive results, and it may be misleading to report kappa alone. Byrt et al. [44] have shown how kappa may be decomposed into components reflecting observed agreement, bias and prevalence, and they proposed a prevalence- and bias-adjusted kappa (PABAK) coefficient that was applied in the following. For comparison with existing literature, the weighted kappa is reported as well. Analyses were performed with SAS v9.4.

For the mixture model analyses on the subscale level for answering research question 2a, latent profile analyses (LPA) were performed with the program LatentGOLD Version 6.1 [45] and the subscale values were treated as ordinal-fixed. The analyses on the item level (research question 2b) were accomplished with latent class analysis (LCA) using the same program. For each subscale, the five items of the child and the five items of the parent were combined and a LCA with these 10 items was performed. To decide on the number of profiles respective classes the Bayesian Information Criterion (BIC) and the Consistent Akaike Information Criterion (CAIC) were evaluated in line with substantive issues regarding the interpretability of the profiles and sufficient class sizes [40]. Each response pattern was assigned to the class with the highest probability. Class labels were assigned descriptively based on the specific response patterns of the latent profiles and the relative levels of the respective mean scores. Differences in means were analysed with paired-sample *t-*tests and Cohen’s *d* was used to provide effect sizes for group differences. For the supplementary analyses regarding age and sex differences between classes, chi²-tests and Cramer’s V were used for sex differences and one-way analysis of variance (F-tests and η²) for age differences.

To test the patterns of agreement between children and parents (research question 3), three different ways of combining the latent class (LC) solutions over the five subscales were explored. First, cross-tabulations between each pair of two LC solutions were examined and the chi²-value and Cramer’s V were used to detect significant associations. If specific cells of the frequency table have higher (or lower) frequencies than expected, chi² and V should be high. This approach is limited because it is only based on pairwise cross-tables and the corresponding meaning of the classes is not taken into consideration. Second, a configural frequency analysis (CFA) of patterns of latent classes was performed. CFAs allow for the identification of specific constellations of categorical variables—so-called configurations—that occur significantly more or less often than expected under the assumption of statistical independence among variables [40]. Configurations that appear more frequently than expected are referred to as *types*, whereas those occurring significantly less are labelled *antitypes* [46, 47]. This person-centered approach enables the detection of meaningful response patterns beyond chance. We performed CFAs using the R package confreq version 1.6.1 [48]. Confreq also provides necessary p-values corrected for the number of combinations (Holm correction; these p-values are mostly around 0.001 with 500 combinations and a full output of confreq is provided on request). Since we are interested in specific patterns, we do not perform such tests on statistical significance on all types/antitypes, but give the uncorrected p-values and focus on the frequency of the interesting patterns. Third, a second-order LCA with nominal categories consisting of the latent classes of the first-order LCA was performed, again estimated with LatentGOLD Version 6.1 [45]. This second order LCA provides a probabilistic alternative to the CFA where the manifest number of combinations can increase quickly.

## Results

### Manifest associations between children and parents on subscale- and item-level

Mean-level comparison showed that children generally reported more difficulties than their parents: The total problem score for the children was 9.63 (SD = 4.85) and for the parents 7.10 (SD = 5.18) and the informants were correlated with *r* = .55. Means and standard deviations at scale and item level and several coefficients of association for the subscales and the items are given in Table 1.

Agreement between parents and children varied across SDQ dimensions: For Emotional Problems, children reported substantially more difficulties than their parents (M = 2.66 vs. 1.70, *d* = 0.45). Overall agreement was modest (r_s_ = 0.42), with the lowest concordance observed for worries (κ(a) = 0.19) and comparatively higher agreement for somatic (κ(a) = 0.52) and feeling unhappy (κ(a) = 0.67). In Peer Problems, children also reported more difficulties than their parents (M = 1.98 vs. 1.36, *d* = 0.39). Agreement at scale level was moderate (r_s_ = 0.48), with particularly high values for having a good friend (κ(a) = 0.76) and being bullied (κ(a) = 0.78), indicating stronger alignment on concrete and externally observable peer experiences. In terms of Hyperactivity/Inattention, the mean scores for both informants were higher than in the other SDQ subscales. Here too, the children’s scores were higher than those of their parents (M = 3.40 vs. 2.52, *d* = 0.43). Agreement at scale level was moderate to high and the agreement at item level relatively consistent across items (r_s_ = 0.53; κ(a) = 0.32–0.40). Regarding Conduct Problems, mean ratings were comparable between both informants (M = 1.60 vs. 1.52, *d* = 0.05), with moderate overall agreement (r_s_ = 0.42). Interestingly, the different indices sometimes led to distinct conclusions. While correlation-based measures generally converged, prevalence- and bias-adjusted kappa values (PABAK) indicated very high agreement for some low-frequency behaviors (e.g., κ(a) = 0.87 for stealing; κ(a) = 0.78 for fighting) compared to Cohen’s kappa, suggesting that high κ(a) values may reflect consistent non-endorsement rather than shared observation of problem behaviors. Finally, Prosocial Behavior showed nearly identical ratings between children and parents (M = 8.11 vs. 8.04, *d* = 0.04) and agreement was modest (r_s_ = 0.42; κ(a) = 0.41–0.61).

Overall, parent–child agreement seemed highest for observable behaviours and lowest for internal or evaluative traits. Differences between correlation-based measures (Spearman’s and Pearson’s r) and prevalence- and bias-adjusted kappa values (PABAK) highlight the influence of item prevalence and response bias on agreement estimates and underscore the importance of employing multiple metrics when assessing cross-informant correspondence.

**Table 1:** Descriptive statistics for children and parents and coefficients of agreement on subscale- and item-level

Subscales / Items (#)	Children M (SD)	Parents M (SD)	chi² (df = 4)	V	*r*_s_	*r*	κ(w)	κ(a)
**Emotional Problems**	**2.66 (2.17)**	**1.70 (1.91)**			**0.422**	**0.464**		
Somatic (3)	0.41 (0.62)	0.46 (0.63)	1433.3	0.37	0.47	0.48	0.42	0.52
Worries (8)	0.80 (0.74)	0.32 (0.55)	423.0	0.20	0.28	0.27	0.16	0.19
Unhappy (13)	0.25 (0.52)	0.16 (0.42)	666.3	0.25	0.34	0.33	0.29	0.67
Clingy (16)	0.69 (0.71)	0.46 (0.63)	440.3	0.21	0.27	0.27	0.21	0.28
Fears (24)	0.50 (0.66)	0.30 (0.54)	532.4	0.23	0.30	0.30	0.24	0.42
**Peer Problems**	**1.98 (1.65)**	**1.36 (1.66)**			**0.478**	**0.541**		
Solitary (6)	0.62 (0.65)	0.39 (0.61)	734.0	0.27	0.34	0.35	0.27	0.35
* Good friend (11)*	0.14 (0.37)	0.15 (0.43)	617.2	0.24	0.30	0.31	0.28	0.76
* Popular (14)*	0.58 (0.59)	0.30 (0.50)	698.1	0.26	0.33	0.34	0.25	0.38
Bullied (19)	0.15 (0.42)	0.17 (0.42)	1162.7	0.33	0.45	0.44	0.41	0.78
Better with adults (23)	0.49 (0.61)	0.35 (0.58)	863.5	0.29	0.36	0.37	0.31	0.45
**Hyperactivity/Inattention**	**3.40 (2.13)**	**2.52 (2.15)**			**0.527**	**0.548**		
Restless (2)	0.58 (0.68)	0.29 (0.55)	712.2	0.26	0.36	0.35	0.26	0.39
Fidgety (10)	0.58 (0.67)	0.19 (0.47)	637.6	0.25	0.32	0.33	0.20	0.36
Distractible (15)	0.68 (0.66)	0.71 (0.70)	1164.0	0.33	0.44	0.44	0.36	0.37
* Reflective (21)*	0.71 (0.56)	0.66 (0.58)	677.7	0.25	0.32	0.32	0.28	0.40
* Persistent (25)*	0.83 (0.63)	0.66 (0.63)	556.3	0.23	0.30	0.29	0.25	0.32
**Conduct Problems**	**1.60 (1.32)**	**1.52 (1.49)**			**0.424**	**0.450**		
Tempers (5)	0.51 (0.64)	0.44 (0.61)	844.7	0.28	0.37	0.38	0.32	0.43
* Obedient (7)*	0.71 (0.55)	0.59 (0.57)	374.3	0.19	0.26	0.25	0.22	0.37
Fights (12)	0.07 (0.27)	0.13 (0.37)	70.6	0.08	0.11	0.10	0.10	0.78
Lies (18)	0.22 (0.48)	0.34 (0.54)	436.7	0.20	0.27	0.28	0.23	0.53
Steals (22)	0.09 (0.32)	0.03 (0.21)	287.4	0.17	0.23	0.21	0.18	0.87
**Prosocial Behavior**	**8.11 (1.65)**	**8.04 (1.70)**			**0.421**	**0.424**		
Considerate (1)	1.65 (0.49)	1.54 (0.55)	294.7	0.17	0.24	0.24	0.21	0.41
Shares (4)	1.64 (0.53)	1.60 (0.56)	383.0	0.19	0.25	0.25	0.23	0.44
Caring (9)	1.77 (0.45)	1.77 (0.46)	388.5	0.19	0.26	0.25	0.25	0.61
Kind to kids (17)	1.71 (0.50)	1.79 (0.45)	385.7	0.19	0.26	0.26	0.24	0.57
Helps out (20)	1.34 (0.56)	1.34 (0.58)	680.3	0.26	0.34	0.34	0.30	0.42

Note: Items in italic are reverse scored; all chi²-values are significant at *p* < .001; *r*_s_ = Spearman *r*; *r* = Pearson *r*; *V* = Cramer’s *V*; κ(w) = weighted Cohen’s kappa; κ(a) = prevalence- and bias-adjusted kappa (PABAK)

### Latent profile analysis at scale level

We conducted a latent profile analysis (LPA) using scale means across the five dimensions of the SDQ as reported by both parents and children. Identifying the optimal number of latent profiles proved challenging, as neither the BIC nor the CAIC reached a clear minimum up to the 11-profile solution (see supplement 1, Figure S4). In light of this ambiguity, we selected a 6-profile solution. This decision was informed by previous findings, particularly the study by Vugteveen et al. [19], who also identified six distinct profiles in this context. The 6-profile solution offers a balance between model parsimony and interpretability, while facilitating meaningful comparisons with existing research.

**Fig. 1:**
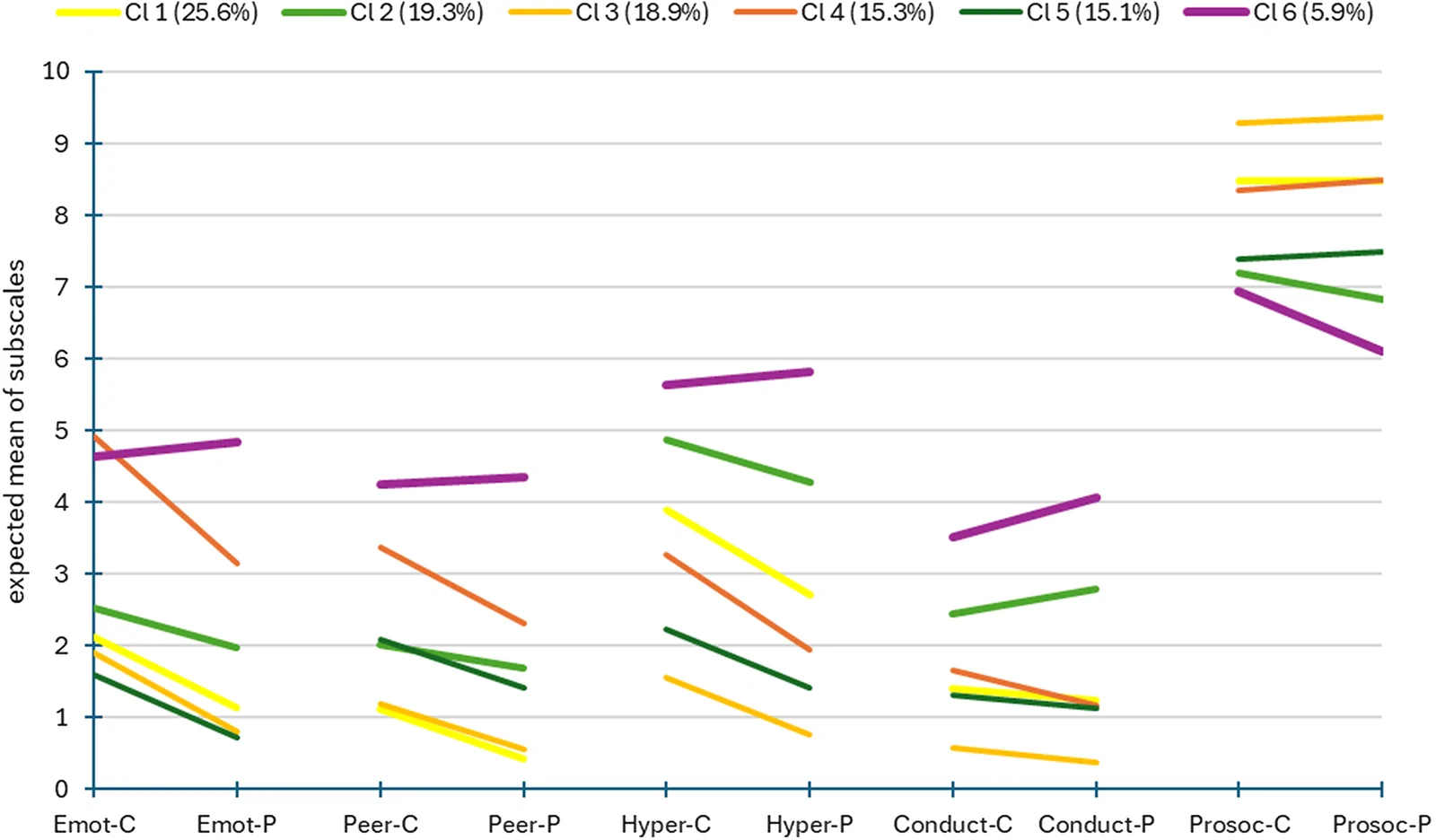
Expected mean values for subscales based on a latent profile analysis with six classes. Note: Subscales are ordered pairwise, first rating of the child (-C), then rating of the parent (-P); Emot = Emotional Problems, Peer = Peer Problems, Hyper = Hyperactivity/Inattention, Conduct = Conduct Problems, Prosoc = Prosocial Behavior

Figure 1 displays the results of the LPA, which was based on the mean scores of the five SDQ subscales, as reported separately by children (-C) and their parents (-P). Note that Prosocial Behavior is a positive trait, so higher scores indicate strengths rather than difficulties, and it is therefore interpreted in the opposite direction compared to the other subscales. All mean values, differences and effect sizes between the two informants are displayed in the supplement 1 (Table S1).

Overall, a general pattern emerges: children tend to report higher levels of difficulties than their parents, particularly on the Emotional Problems, Peer Problems, and Hyperactivity/Inattention subscales (see also Table 1). This discrepancy is evident in the profiles of Clusters 1, 3, 4, and 5. Cluster 2 shows only minor differences between child and parent reports, mainly on Hyperactivity/Inattention. Cluster 6 stands out as the only group in which parents report (slightly) higher symptom levels than the children—this cluster is also the most symptomatic overall. Cluster 3 shows the highest levels of Prosocial Behavior, while Cluster 6 is lowest, with some disagreement between informants.

To sum up, the six clusters can be interpreted as follows:

- Cluster 1: “Low internalizing with child–parent discrepancy” (25.6%).​
  Low internalizing symptoms and medium hyperactivity; moderate disagreement, with children reporting more symptoms than parents in all subdimensions (labelled: C > P).
- Cluster 2: “Elevated externalizing with agreement” (19.3%).
  Low internalizing symptoms and high hyperactivity and conduct problems; close alignment between child and parent ratings.
- Cluster 3: “Low symptoms with slight disagreement” (18.9%).
  Largely asymptomatic profile and highest prosocial scores; some child–parent discrepancy.
- Cluster 4: “High internalizing and externalizing with disagreement” (15.3%).
  High internalizing symptoms and elevated externalizing symptoms; notable disagreement, with children reporting more problems in all subdimensions (C > P).
- Cluster 5: “Slightly elevated with moderate disagreement” (15.1%).
  Slightly more symptoms than Cluster 3 across domains; moderate discrepancy between child and parent ratings.
- Cluster 6: “High symptom burden with agreement” (5.9%).
  High scores across all difficulty domains and lowest prosocial behavior; child and parent reports are closely aligned.

The differences between child and parent means, calculated by Cohen’s *d*, are small to medium (0.40–0.71) for Clusters 1, 3, 4 and 5 for the subscales Emotional Problems, Peer Problems and Hyperactivity/Inattention whereas in Cluster 2 and 6 they are small (-0.13–0.25). In contrast, the subscales Conduct Problems and Prosocial Behaviour show mostly small to neglectable *d*s for all Clusters (see supplement 1, Table S1). Age means and gender percentages in relation to the identified clusters of parent–child agreement and disagreement are displayed in the supplement 1 (Table S3).

### Latent class analysis at item level

**Fig. 2:**
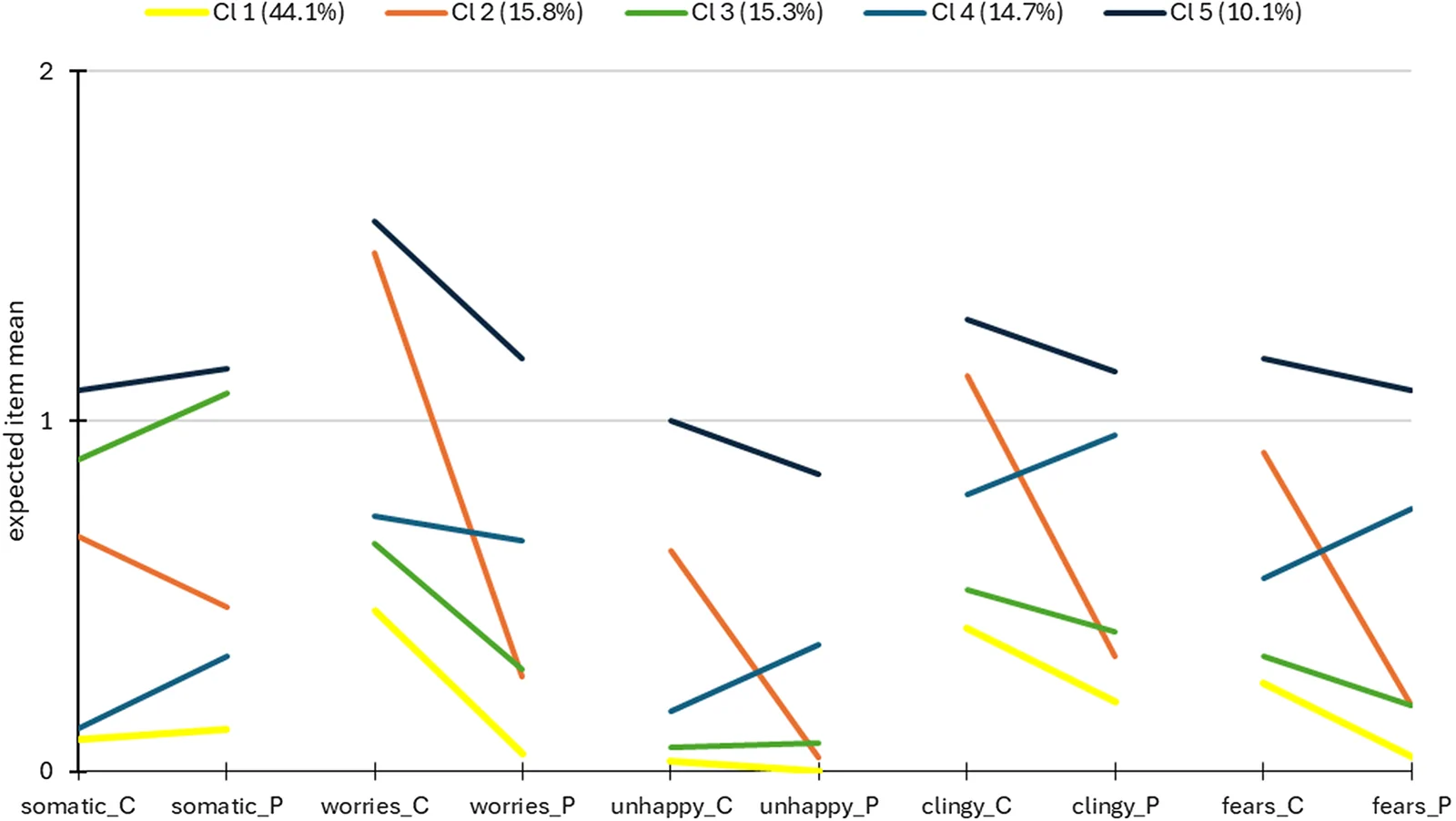
Expected item means for the 5-class solution for the subscale Emotional Problems. Note: Items are ordered pairwise, first rating of the child (_C), then rating of the parent (_P)

**Fig. 3:**
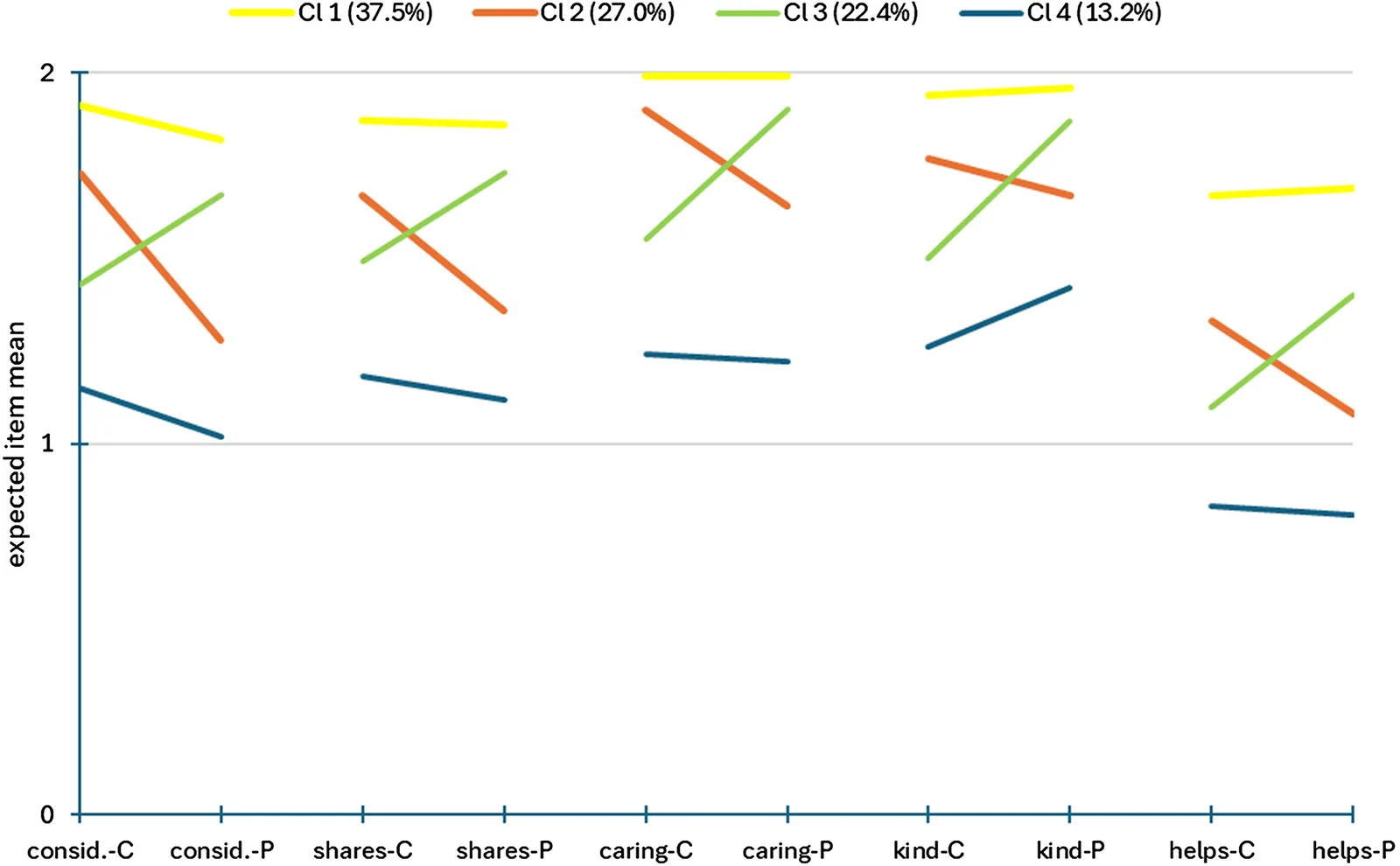
Expected item means for the 4-class solution for the subscale Prosocial Behavior. Note: Items are ordered pairwise, first rating of the child (-C), then rating of the parent (-P)

To further examine symptom patterns and potential discrepancies between informants, we conducted five separate latent class analyses (LCAs) at the item level – one for each SDQ subscale. In each LCA, the five items corresponding to the respective subscale were included, with separate reports from children and parents. Class enumeration was in each case performed by inspecting the so-called elbow plot of the information criteria (ICs). This was ambiguous in all cases (see supplement 2), which is why additional decisions were made based on parsimony and interpretability. Due to space limitations, detailed graphical representations are only presented for the subscales Emotional Problems and Prosocial Behavior in the following (Figs. 2 and 3). The figures for the remaining three subscales are available in the supplement 1 (Figures S1 – S3).

#### Emotional problems

Figure 2 presents the results of the LCA for the subscale Emotional Problems at the item level. Five distinct latent classes were identified, each reflecting characteristic constellations of symptom patterns and informant agreement. The largest group, Class 1, can be described as “low-symptomatic – concordant “, with consistently low item ratings from both children and parents, indicating an absence of notable emotional difficulties. In contrast, Class 5, labelled “high symptomatic – concordant”, comprises cases in which both informants report high levels of emotional problems across all items. Between these two extremes lie three more differentiated profiles: Class 2, referred to as “Child-reported distress – discordant”, is marked by elevated symptom reports from children, while parents report comparatively low levels across all items. Class 4, in turn, represents the opposite pattern and is labelled “Parent-reported concerns – discordant”. In this class parents indicate elevated emotional difficulties, whereas children report few symptoms (with a slight exception on the item worries). Finally, Class 3, termed “high somatic-focused symptoms – concordant”, is characterized by a specific elevation of somatic complaints, while all other emotional symptoms are rated low by both informants.

#### Prosocial behavior

The item-level results of the LCA for the subscale Prosocial Behavior are displayed in Fig. 3. Four distinct classes were identified, reflecting distinct profiles in prosocial behavior and patterns of agreement between child and parent reports.

The largest class, Class 1, can be described as “high prosocial – concordant” and includes children who reported consistently high levels of prosocial behavior, which were closely mirrored by their parents’ ratings. This class reflects a group of well-adjusted children with strong prosocial tendencies and high informant agreement. Class 2, labelled “Child-reported medium prosocial – discordant”, is characterized by high prosocial self-reports by the children, while parents provided more moderate ratings. This pattern indicates a discrepancy in perception, where children see themselves as more prosocial than their parents perceive them to be. In contrast, Class 3, labelled “Parent-reported medium prosocial – discordant”, shows the reverse pattern: parents reported higher levels of prosocial behavior than the children themselves. This may suggest that children underreport their prosocial actions or that parents interpret certain behaviors more positively. Finally, Class 4, labeled “Low prosocial – concordant”, includes children who were rated low in prosocial behavior by both themselves and their parents. This class represents a group with consistently low prosocial engagement as perceived by both informants.

#### Overview of LCA solutions

Table 2 provides an overview of the number of classes identified for each of the five subscale and gives a brief characterization of the resulting latent classes.

**Table 2:** Overview of identified LCA classes per subscale

SDQ Subscale	Number of Classes	Short Description of Identified Classes (Class %)
Emotional Problems	5	1: low-symptomatic – concordant (44.1%) 2: Child-reported distress – discordant (C > P; 15.8%) 3: high somatic-focused symptoms – concordant (15.3%) 4: Parent-reported concerns – discordant (P > C; 14.7%) 5: high symptomatic – concordant (10.1%)
Peer Problems	4	1: Low peer problems – concordant (50.4%) 2: Child-reported mild peer difficulties – discordant (C > P; 26.5%) 3: Moderate peer issues – concordant (16.3%), 4: high parent-reported peer problems – discordant (P > C; 6.8%)
Hyperactivity/Inattention	5	1: Inattentive without hyperactivity – concordant (26.9%) 2: Inattentive and hyperactive – concordant (24.7%) 3: Low symptoms – slightly discordant (C > P, 23.6%) 4: More symptoms reported by child – discordant (C > P; 17.5%) 5: Highly hyperactive – concordant (7.3%)
Conduct Problems	5	1: Irritable and oppositional – concordant (32.8%) 2: Low conduct problems – concordant (28.5%) 3: Disobedient only – concordant (21.3%) 4: Child-reported conduct issues – discordant (C > P; 8.8%) 5: Severe conduct problems – slightly discordant (P > C, 8.7%)
Prosocial Behavior	4	1: High prosocial – concordant (37.5%) 2: Child-reported medium prosocial – discordant (C > P; 27.0%) 3: Parent-reported medium prosocial – discordant (P > C; 22.4%) 4: Low prosocial – concordant (13.2%)

#### Mean differences between children and parents for the LCA solutions

**Fig. 4:**
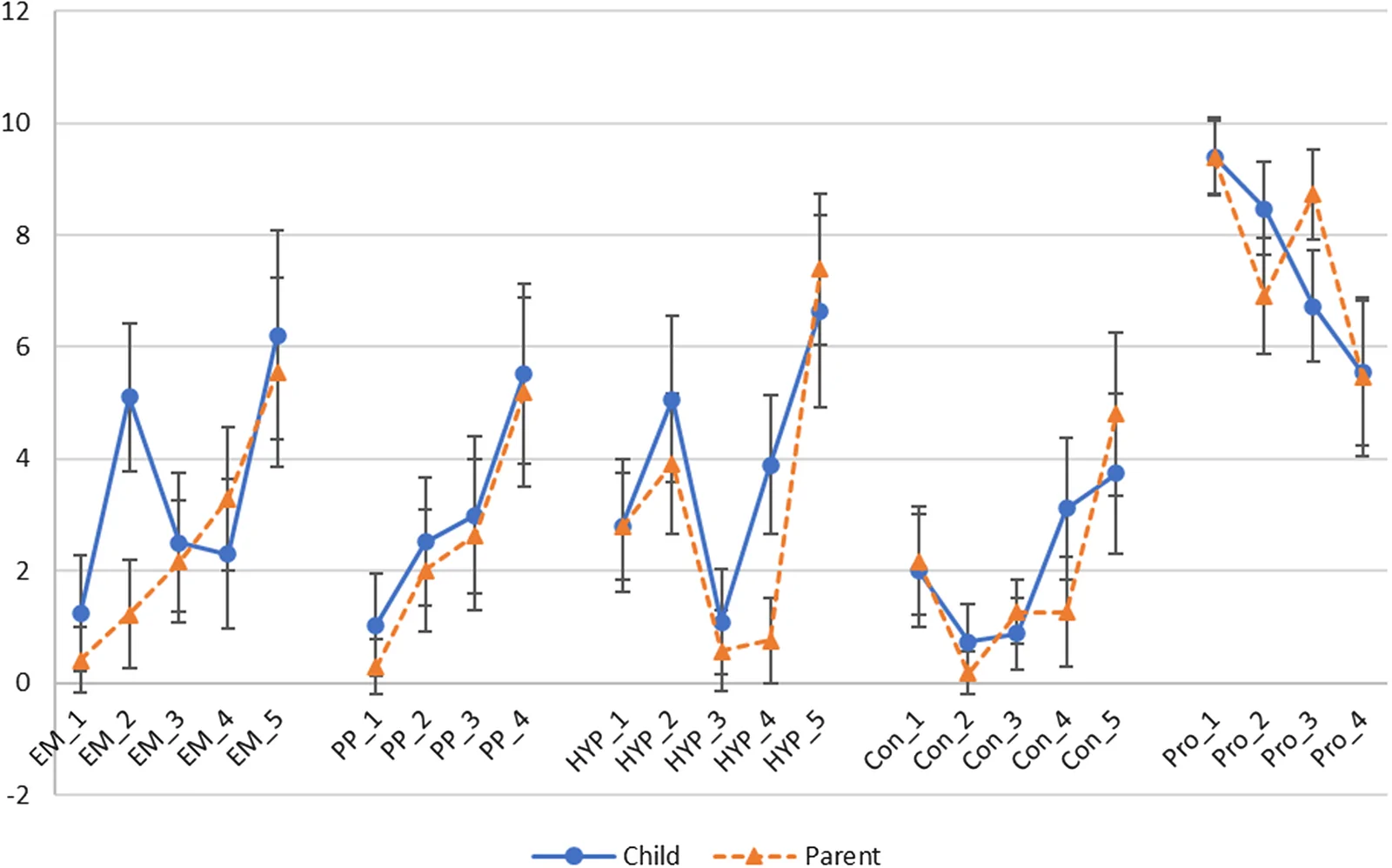
Means and SD (± 1) for each latent class. Note: EM = Emotional Problems, PP = Peer Problems, HYP = Hyperactivity/Inattention, Con = Conduct Problems, Pro = Prosocial Behaviour

Figure 4 displays the means and standard deviations for each latent class for the respective SDQ subscales, separately for child and parent reports. These descriptive values were used to support the interpretation and labelling of the latent classes derived from the LCAs. The figure illustrates that the greatest discrepancies exist in classes EM_2, Hyp_4, Con_4 and Pro_3. Across most classes, children tended to evaluate their own behaviour more negatively compared to their parents. All mean differences were significant at *p* < .0001, except Peer_4 (*p* = .014) and Hyp_1, Pro_1, and Pro_4 (all *p*s > 0.40). Age means and gender percentages in relation to the identified LCA classes are displayed in the supplement 1 (Table S3).

### Associations between latent classes

Based on the previous results, the following paragraphs describe three different ways of combining the LC solutions across the five subscales: Contingency tables, CFA analysis and second order LCA.

#### Contingency tables

Cross-tabulations between each pair of two LC solutions revealed significant chi²-values in all pairs of subscales. The effect size (Cramer’s V), however, was mostly small (V < 0.17). Only the association between the internalizing subscales (Emotional Problems, Peer Problems) on the one hand, and the externalizing subscales (Hyperactivity/Inattention, Conduct Problems) on the other hand is higher than the other combinations and effect sizes were moderate (V = 0.21 and 0.24, respectively). Full results of this approach can be found in the supplement 1 (Table S2).

#### Configural frequency analysis (CFA) of patterns of latent classes

CFA uses all available patterns of the latent class solutions which will reach up to 2000 possible combinations for the five subscales. Thus, we focused on the four problem subscales which theoretically provided 500 different patterns (5 × 4 × 5 × 5). The frequency of being always in the low-symptomatic class (pattern: 1 1 3 2) included 307 child-parent pairs and emerged clearly as a type (z = 20.40, *p* < .0001). Analogously, the pattern of being always in the high-symptomatic class (5 4 5 5) was significantly found as a type (z = 47.10, *p* < .0001, *n* = 20), and minor variations of this pattern (e.g., 5 3 5 5) also showed significantly higher frequencies than expected. The most interesting pattern where the pairs were in the “child > parent” (C > P) class across all four subscales (2 2 4 4) included only four pairs which was not significantly different from the expected frequency (z = 1.08, *p* = .140). Thus, the initially hypothesized pattern where children rated consistently higher than the parent across all four subscales was neither significant nor relevant in frequency. The opposite pattern (“parent > child”) was less pronounced and can be approximated by 4 4 × 5, where X means there is no P > C Class in Hyperactivity/Inattention. Therefore, we looked at all patterns where Hyperactivity/Inattention is not C > P (4 4 1 5, 4 4 2 5, 4 4 5 5). The last two of them are significant types (z = 9.37, *p* < .0001, *n* = 10; z = 17.31, *p* < .0001, *n* = 9) but small in size.

As a supporting analysis, we looked at the class combinations of the internalizing subscales (Emotional Problems and Peer Problems) on the one hand, and externalizing subscales (Hyperactivity/Inattention and Conduct Problems) on the other hand. The combination of being in the low-symptomatic class in both subscales was highly significant for the internalizing as well as the externalizing two subscales. The same was true for the combination of being in the high-symptomatic in both subscales. The most interesting combination of “child > parent” (pattern: 2 2) revealed no significantly increased frequency (z = 0.98, *p* = .162, *n* = 231) for the two internalizing subscales. The frequency for the same combination in the two externalizing subscales (pattern: 4 4) was significant (z = 2.32, *p* = .010, *n* = 74).

Summarizing, there is some hint on a “type” for the C > P in the two externalizing subscales, but the practical relevance of this finding seems small. For the two internalizing subscales, no increased combination was found and for the total of all four subscales, there is obviously no consistent “children > parent” subgroup. Post hoc inspection of the mean values in Fig. 4 shows the highest differences between child and parent ratings in classes EM_2 and HYP_4. Analysis of this combination confirmed a significantly increased frequency (z = 5.27, *p* < .001, *n* = 188). Further exploratory analyses with the grouping option of the package confreq revealed that girls are significantly found more often in this combination than boys (z = 25.41, *p* < .0001).

#### Second order latent class analysis

**Fig. 5:**
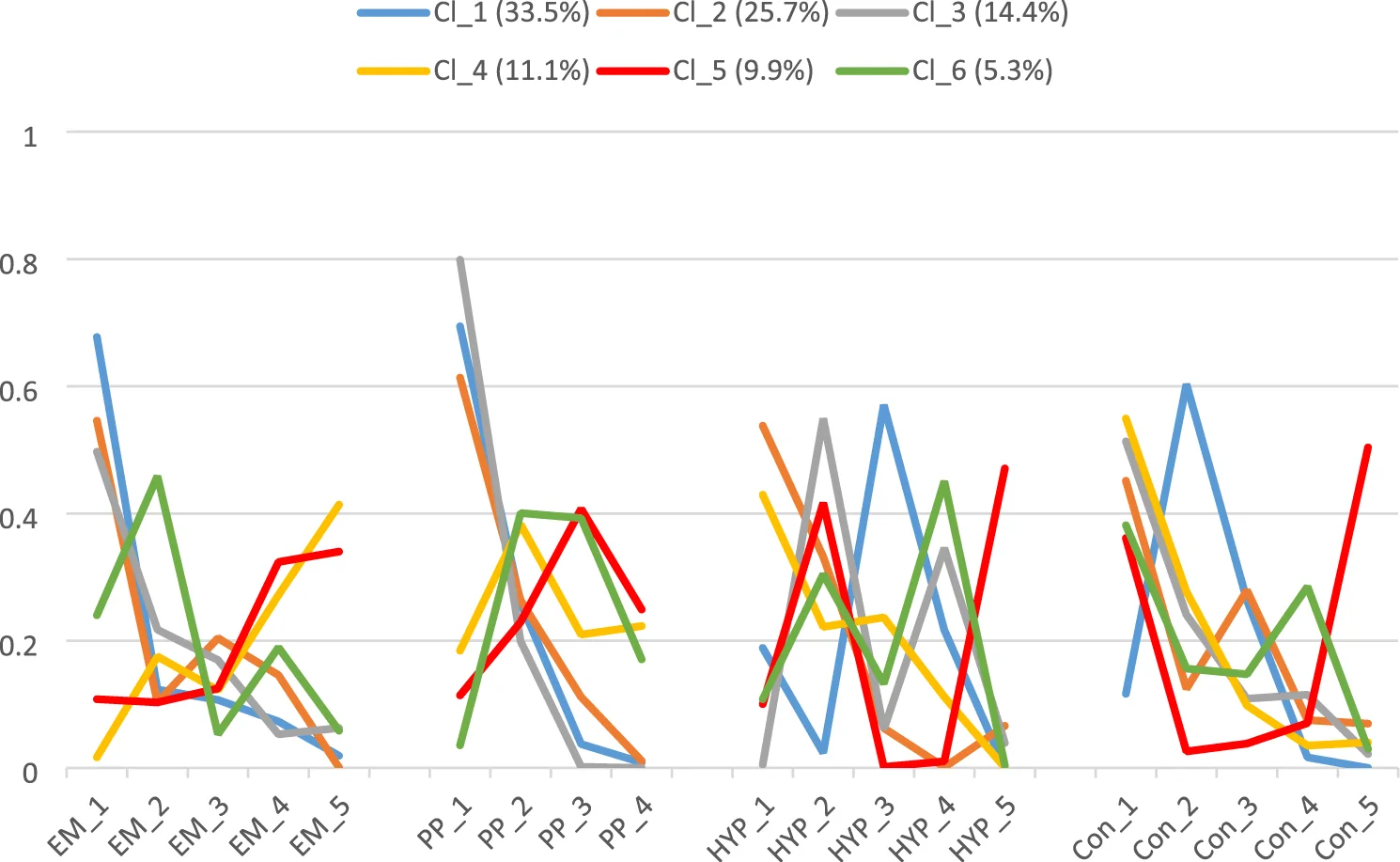
Expected item means for the 6-class solution of the second order LCA. Note: EM_1 etc. represent the latent classes as shown in Table 2

The identification of the optimal number of latent classes suggested a four-class solution based on the BIC and CAIC, both of which reached a minimum at this level. However, we opted for a six-class solution, as it provided a more differentiated and interpretable representation of the data and the BICs increased only slightly for more classes. In particular, the six-class solution allowed for the identification of two additional, substantively meaningful subgroups that were highly relevant to our research focus on patterns of agreement and disagreement between child and parent reports. The six-class solution (see Fig. 5) therefore offered a better balance between statistical fit and substantive interpretability and is presented in the following:

- Class 1 “Well‑Adjusted Concordant Group” (33.5%): Children in this class predominantly belonged to the non-symptomatic, concordant subtypes across all domains (e.g., EM_1, PP_1, HYP_3, CON_2). This pattern reflects consistently low symptom levels and high agreement between parent and child reports, indicating a well‑adjusted and concordant group.
- Class 2 “Mild Externalizing Concordant Group” (25.7%): This class exhibited generally low emotional and peer problems (EM_1, PP_1) alongside mild attentional and behavioral difficulties, particularly inattentiveness without hyperactivity (HYP_1) and irritable/oppositional behavior (CON_1). It represents children with mild, selective externalizing tendencies who show concordance between reporters.
- Class 3 “Pronounced Externalizing Concordant Group” (14.4%): Members of this class showed low emotional and peer difficulties (EM_1, PP_1) but elevated hyperactivity (especially inattentive and hyperactive, HYP_2; and partly child-reported hyperactivity, HYP_4) and a strong concentration in irritable/oppositional conduct problems (CON_1). Compared with Class 2, this pattern reflects a stronger and more consistent externalizing profile marked by pronounced hyperactivity and oppositional behavior.
- Class 4 “Heterogeneous Cross‑Domain Concordant Difficulty Group” (11.1%): This class displayed a mixed and moderately elevated pattern across domains, with probabilities distributed among emotional and peer difficulties (EM_5, PP_2/3/4) and conduct problems (CON_1), but without concentration in the most severe subgroups. It represents a heterogeneous group with moderate, cross-domain difficulties.
- Class 5 “Parent‑Reported Difficulty (P > C) Group” (9.9%): Children in this class were predominantly assigned to discordant, problem‑oriented subtypes with higher parent‑reported symptoms across multiple domains (e.g., EM_4/5, PP_3/4, CON_5), except hyperactivity. This indicates parent–child disagreement where parents perceive more difficulties.
- Class 6 “Child‑Reported Difficulty (C > P) Group” (5.3%): This class showed elevated probabilities of higher child‑reported symptoms across multiple domains—particularly emotional and peer problems (EM_2, PP_2/3), hyperactivity (HYP_4), and to a lesser extent conduct issues (CON_1/4). Compared with Class 5, the symptom burden is somewhat lower and more domain‑specific, reflecting a child‑reported difficulty pattern with cross-domain disagreement (C > P).

## Discussion

In this study, we investigated child – parent agreement on the Strengths and Difficulties Questionnaire (SDQ) in a nationally representative German sample from the second wave of the German Health Interview and Examination Survey for Children and Adolescents (KiGGS). Using both manifest and latent analytical approaches, we examined the structure and degree of agreement between children’s and parents’ reports across all five SDQ subscales. Specifically, we assessed (1) the correlational patterns of manifest SDQ scores and items, (2) latent discrepancies in parent and child ratings on scale- and item-level, and (3) the existence of distinct subgroups characterized by consistently high or low agreement across mixture models for all subscales. By combining correlational, item-level, and person-centered analyses, this study provides a comprehensive picture of the convergences and divergences between parent and child perspectives on children’s behavioral difficulties and strengths.

Concerning reliability, Cronbach’s Alpha was acceptable for the subscales Emotional Problems and Hyperactivity/Inattention.​ In contrast, lower reliabilities were observed for the subscales Peer Problems, Prosocial Be​​havior and especially Conduct Problems. This pattern is consistent with previous research [15, 22, 34, 49] showing comparatively modest internal consistency for some SDQ domains, particularly for the subscales Conduct Problems and Peer Problems. While these limitations should be considered when interpreting the results, the SDQ remains a widely used screening instrument, and similar reliability patterns have been reported in other population-based studies.

### Manifest associations between children and parents

Overall, agreement between parent and child SDQ subscale ratings was modest. Agreement was highest for the Hyperactivity/Inattention and Peer Problems subscales, suggesting that parents and children show relatively consistent perceptions of attentional and peer-related difficulties.

Correlation coefficients between child and parent report were found to be in the range of *r* = .42 − .55 for the subscales and *r* = .55 for the TDS. These values were higher than in other studies (e.g [28]), and the current values significantly exceed the aggregated correlation coefficients from the meta-analysis of Bergström and Baviskar ([30]; range of *r* = .30. − 0.37 for the subscales and *r* = .46 for the TDS) for community samples.

On the item-level, the prevalence- and bias-adjusted kappas (PABAK) reveal that, out of the 25 SDQ items, 17 (68%) had perfect to moderate agreement (4% almost perfect; 24% substantial; 40% moderate), and 8 (32%) had fair to slight agreement (28% fair; 4% slight) whereas the PABAK values of Liu et al. [23] are mostly lower: 9 items (36%) had perfect to moderate agreement (8% almost perfect; 16% substantial; 12% moderate), 15 (60%) had fair to slight agreement (40% fair; 20% slight), and 1 (4%) had agreement less than chance.^1^

Similar to Liu et al. [23] the items of the subscale Prosocial Behaviour have the highest PABAKs. Within the subscale Conduct Problems, the item steals reveals an almost perfect agreement (PABAK = 0.87) which points to more consistency between parents and children if certain deviant behaviors are known. A consistently low proportion of agreement reveals the subscale Hyperactivity/Inattention (PABAK between 0.32 and 0.40), but on the subscale level, the correlation was the highest. The lowest agreement was found in two items of the subscale Emotional Problems (items worries: 0.19 and clingy: 0.28). These results do not only point to differences in the knowledge but also may indicate a different understanding of the items between parents and children. The item somatic revealed the highest kappa in the subscale Emotional Problems as found already in Keller et al. [31], but the adjusted kappa (PABAK) pointed to the item unhappy as the item with the highest agreement. The item unhappy had a low prevalence and therefore, the difference between the two coefficients gets larger, and most obvious in the conduct items steals and fights. However, PABAK is one of several methods to assess agreement and does not provide the “true” value. Thus, it is recommended to use multiple methods and to be aware that the coefficients describe different aspects of agreement [50]. Nonetheless, the wide range of agreement coefficients across SDQ items indicates that child–parent agreement is highly item-specific and cannot be adequately captured by subscale-level agreement alone.

### Latent profile analysis and latent class analysis

The latent profile analysis (LPA) identified six distinct profiles of child mental health based on parent and child SDQ reports. Profiles differed both in overall symptom severity and in the level of child–parent agreement. Notably, Clusters 1 and 4 were characterized by discrepancies in which children reported higher symptom levels (C > P) than their parents across all difficulty domains. In contrast, no profile showed a systematic pattern of parents reporting more problems than children (P > C). Comparing Clusters 1 and 4 further illustrates that these discrepancies occur at different levels of overall symptom severity. Cluster 1 represents a relatively low-risk profile with generally low levels of difficulties, whereas Cluster 4 is characterized by elevated internalizing and externalizing symptoms. Importantly, symptom levels in Cluster 1 were consistently lower than in Cluster 4 across domains, with the exception of Hyperactivity/Inattention, which appeared comparatively pronounced even within the otherwise low-symptom profile.

The item-level LCAs further underscored the heterogeneity of symptom patterns within each SDQ domain and revealed systematic structures in informant agreement and disagreement. Across all subscales, classes reflecting concordant low symptom levels emerged as the largest groups, indicating that a substantial proportion of children are consistently perceived as well-adjusted by both informants. At the same time, each domain also showed symptomatic classes with high agreement, suggesting that more pronounced difficulties are generally recognized similarly by children and parents. Beyond these concordant patterns, the analyses consistently identified systematic discrepancy classes, in which either children or parents reported higher levels of problems. Notably, child-greater-than-parent patterns were more frequently observed across all domains, indicating that children often perceive more difficulties than are reported by their parents. Conversely, parent-greater-than-child discrepancies also emerged but tended to be less prevalent and more domain-specific.

Importantly, the item-level analyses revealed that discrepancies are not uniform across all symptoms but may be item-specific or domain-specific, as illustrated by profiles such as somatic-focused emotional problems or selective difficulties in attention and behavior. Methodologically, the broad variety of latent classes identified across the different SDQ subscales suggests that the LCA did not simply reproduce the same agreement or discrepancy patterns across domains. Rather than reflecting methodological artifacts, these findings point toward genuinely domain-specific constellations of perception and informant agreement, indicating that child–parent discrepancies may vary substantially depending on the specific domain.

Our findings align with previous person-centered research using latent class or latent profile approaches to examine informant discrepancies. For example, Makol et al. [37] identified by analysing three internalizing scales (anxiety, depression, somatization) of the Behavior Assessment System for Children (BASC) four adolescent–parent classes reflecting concordant low and high symptom reports as well as discrepant constellations (adolescent > parent and parent > adolescent), thereby contributing to a better understanding of how multi-informant reports can inform the interpretation of adolescents’ clinical presentations and predict treatment-related characteristics. Similarly, Vugteveen et al. [19] reported six SDQ-based profiles indicating different types and combination of difficulties. They concluded that combining the information from both informants was superior in prediction, but parent reports may provide the more informative source of assessment when only a single informant can be used. The study of Vugteveen et al. [19], however, had a clear focus on prediction of diagnoses by single vs. multi-informant SDQ data, and potential discrepancies of the subscale means between the two informants were not further evaluated. Keller et al. [31] identified both concordant and systematically discrepant classes, particularly for the SDQ subscales Emotional Problems and Prosocial Behavior, where discrepancies were substantial. Our results largely replicate the patterns reported in previous person-centered studies by identifying profiles characterized by concordant ratings and profiles in which children report more difficulties than their parents.

### Associations across domains

The findings of the pairwise contingency tables suggest that, while there is some overlap in class membership across subscales, the associations are generally weak, indicating largely domain-specific patterns of symptom expression; accordingly, they do not support the presence of overarching, cross-domain discrepancy classes. However, the somewhat stronger links between internalizing and externalizing domains point to a modest but meaningful co-occurrence of broader problem constellations. In particular, the combination of Emotional Problems and Hyperactivity/Inattention showed the strongest discrepancies, suggesting a more pronounced co-occurrence of internalizing and externalizing difficulties; these cross-domain patterns are examined in detail in the subsequent configural frequency analyses.

The aggregation of all four subscale-specific LCA solutions (CFA) also did not reveal a consistent subgroup characterized by a C > P assessment. Accordingly, our initial assumption of a generalized cross-domain discrepancy pattern must be rejected. While the two internalizing subscales (Emotional Problems and Peer Problems) did not show an increased frequency of C > P constellations, such a pattern emerged more clearly within the externalizing domains (Hyperactivity/Inattention and Conduct Problems). Moreover, a significant association between specific and large discrepancy classes (EM_2 and HYP_4) suggests that child-dominant reporting (or parental non-awareness) patterns may still co-occur across selected domains, providing some partial support for the existence of a C > P subgroup. However, these findings should be interpreted with caution and require replication in future studies. One possible explanation for the lack of a consistent cross-domain pattern may lie in the relatively strict nature of the CFA-based approach. In contrast, more flexible, probabilistic approaches such as the second-order LCA may be better suited to capture heterogeneous and domain-specific patterns of agreement and disagreement between informants. In fact, the 500 possible patterns of the CFA were reduced to six latent classes in the second order LCA.

The second-order LCA revealed six distinct cross-domain subgroups that differed not only in overall symptom burden but also in systematic patterns of agreement and disagreement between child and parent reports. Notably, about one third of the sample was consistently assigned to a low-symptomatic class with high convergence between both informants, indicating a broadly well-adjusted group (Class 1). Beyond this, several classes reflected more differentiated patterns of externalizing difficulties and cross-domain impairment. Importantly, two distinct subgroups (Class 5 and 6) emerged that were characterized by systematic informant discrepancies, with either parents or children consistently reporting higher levels of problems across domains. These findings suggest that child–parent agreement is not uniform but follows structured patterns, indicating that discrepancies are not merely random measurement error.

Similar to earlier LCA approaches, we identified distinct groups reflecting concordant low and high symptom reporting as well as opposing child–parent perspectives. At the same time, extending prior work, our second-order LCA highlights that these patterns of agreement and disagreement are embedded within broader cross-domain constellations of symptoms. In this regard, our results complement findings from LPA studies on SDQ profiles [19] by showing that heterogeneity is not only present in symptom severity and domains, but also in the degree and direction of informant agreement across domains.

### Integrative discussion and implications for subscales

Taken together, the findings across the manifest and latent analyses provide a differentiated picture of child–parent agreement on the SDQ and demonstrate that agreement patterns depend strongly on both the level of analysis and the specific subscale of the SDQ. While the overall results are complex, several overarching conclusions can be drawn with regard to the three research questions:

Concerning research question 1, the manifest analyses showed moderate associations between child and parent reports across SDQ subscales, with agreement coefficients generally exceeding those reported in previous community-based studies. At the same time, agreement proved to be highly item-specific. Particularly on the item level, substantial variability emerged not only between subscales but also within the same subscale, indicating that some symptoms are perceived much more similarly by children and parents than others. These findings suggest that important nuances of child–parent agreement remain obscured when analyses rely solely on aggregated subscale scores.

With respect to research question 2, both the scale-level LPA and the item-level LCAs identified meaningful latent subgroups characterized by varying levels of agreement and discrepancy between informants. At the scale level, the LPA identified two profiles marked by systematic C > P patterns, whereas no clear P > C profile emerged. In addition, profiles characterized by close alignment and profiles with more selective discrepancies were identified, indicating that disagreement occurs at different levels of symptom severity and across different constellations of behavior problems.

The item-level LCAs further refined these findings by demonstrating that discrepancy patterns vary considerably across domains. Across all problem subscales, a distinct C > P class emerged, although the magnitude and clarity of this pattern differed between domains. P > C patterns were also identified, but tended to be less pronounced, smaller in size, and absent in the subscale Hyperactivity/Inattention. Interestingly, the clearest and most symmetrical discrepancy patterns were observed in the subscale Prosocial Behavior, where the analyses yielded the four theoretically expected classes in a particularly “pure” form: high concordant, low concordant, child-greater-than-parent, and parent-greater-than-child. This finding may indicate that positively framed behaviors are especially sensitive to differences in self- versus parent perception.

Across both latent approaches, two robust patterns consistently emerged: first, a large concordant low-symptom group, and second, a smaller but clearly identifiable concordant high-symptom group. This suggests that agreement between children and parents may be strongest at the extremes of symptom severity, whereas discrepancies are more likely to emerge in intermediate or ambiguous symptom constellations. Notably, these patterns became particularly visible in the latent analyses and were much less apparent at the manifest level, underscoring the added value of mixture modelling approaches for studying informant agreement.

Regarding research question 3, the findings provide only limited support for the existence of a generalized cross-domain discrepancy type. The CFA-based aggregation of latent classes did not identify a subgroup characterized by consistently elevated child reports across all four SDQ problem scales. Thus, the assumption of a uniform cross-domain C > P pattern could not be confirmed. However, the second-order LCA identified a small subgroup characterized by systematic C > P discrepancies across multiple domains, alongside a corresponding but even smaller P > C subgroup. These findings are in line with Keller et al. [31] and suggest that broad discrepancy patterns may exist, but likely in a probabilistic rather than deterministic manner and small in size. The second-order LCA appeared particularly useful in this regard, as it allowed for more flexible and heterogeneous combinations of agreement patterns across domains than the more restrictive CFA-based approach.

At the level of individual subscales, several noteworthy patterns emerged: For Emotional Problems, agreement structures were particularly heterogeneous. While concordant low- and high-symptom classes emerged, one especially pronounced discrepant class (EM_2) was characterized by substantially higher child-reported distress. In addition, agreement varied strongly across items, with comparatively low agreement for worries and clingy, similar to the results of Liu et al. [23]. These differences may partly be attributable to variations in item wording between the child and parent versions as well as differences in symptom visibility or interpretation. Future studies should therefore examine whether such discrepancy patterns vary systematically by characteristics such as age or gender.

For Peer Problems, agreement coefficients were comparatively modest and mean-level differences between informants remained relatively small. Although the analysis identified four classes with one characterized by higher C > P reported peer difficulties, these discrepancies were generally small in magnitude and did not reflect markedly distinct qualitative response patterns across profiles. Rather, children tended to rate their peer difficulties consistently only slightly higher than their parents across all classes. This comparatively uniform pattern suggests that, for peer problems, the mixture modelling approach yielded limited additional insight beyond the overall mean-level differences between informants.

In contrast, Hyperactivity/Inattention showed one of the clearest discrepancy patterns, particularly in the HYP_4 class, where children consistently reported substantially more symptoms than their parents suggesting systematic perception differences that are not immediately visible at the manifest scale level. A similar pattern emerged for Conduct Problems, especially in the CON_4 class. These findings may indicate that attentional and behavioral regulation problems are more salient to children themselves than to parents in certain subgroups. Again, future research should investigate which child- or family-related factors are associated with these discrepancy profiles.

Finally, Prosocial Behavior showed perhaps the clearest latent structure of all subscales. Here, the analyses identified two highly concordant classes (high and low prosocial) as well as two almost perfectly opposing discrepant classes, with either children or parents reporting systematically higher prosocial behavior. The particularly clear separation of these classes suggests that positively framed behaviors may be especially informative for studying systematic informant discrepancies.

The supplementary analyses exploring age and sex differences across the identified classes revealed (supplement 1, Table S3) with regard to the Latent Profile Model and the second-order LCA model that the classes showed significant differences by gender and age observed across the respective six classes, but the corresponding effect sizes (Cramer’s V and eta²) were small. Similarly, the five sub-dimensions of the SDQ showed significant differences between the four or five classes in the LCA models at item level; however, the V-values for gender indicated small effects, and the eta² values for age were also in the range of small effects. Two findings are particularly worth noting: the greatest differences in gender proportions were found in the Emotional Problems subscale; and, as regards minor differences, there was almost no significant gender difference in the Peer Problems subscale (*p* < .05, below a small effect size) and no significant age difference in the Prosocial Behaviour subscale. Due to space limitations, however, these results are not discussed in more depth, but they may help to provide insight for future confirmatory studies.

Overall, our findings have important methodological and practical clinical implications. Methodologically, the results demonstrate that conclusions about child–parent agreement depend substantially on the analytical approach used. Manifest analyses tend to obscure heterogeneity in agreement patterns, whereas latent mixture models are able to uncover meaningful subgroups characterized by specific constellations of convergence and divergence. The item-level LCAs, in particular, revealed that different patterns of agreement can exist within the same subscale, suggesting that agreement should not be evaluated solely at the scale level. This is particularly relevant because SDQ subscales comprise conceptually distinct symptoms and behaviors, and similar subscale scores may therefore mask disagreement regarding specific difficulties. At the individual level, considering such item-specific discrepancies may complement subscale scores by highlighting symptoms that are perceived differently by children and parents. Our findings reinforce the importance of integrating both child and parent perspectives. Discrepancies between informants should not merely be treated as measurement error and analysed by measurement invariance techniques [6] but considered potentially meaningful indicators of contextualized symptom perception, communication processes, or differential symptom visibility [18, 26, 36].

### Limitations and outlook

The present study provides novel insights into systematic patterns of agreement between child and parent reports across all SDQ subscales, extending previous research that has rarely examined all domains simultaneously, particularly in a representative national sample. However, several limitations should be considered. First, while the applied person-centered approaches allowed for the identification of meaningful subgroups, they remain descriptive in nature and do not allow conclusions about causal relationships or underlying mechanisms. Moreover, the present study focuses on patterns of agreement and disagreement between child and parent reports and does not allow conclusions regarding the relative accuracy of either informant. Independent external validators, such as clinical diagnoses, treatment utilization, or indicators of functional impairment, were not available in the KiGGS data used for the present analyses. Linking discrepancy patterns to such external criteria would be an important avenue for future research, as it could help determine whether and under which circumstances child or parent reports correspond more closely to independent indicators of adolescents’ mental health and functioning. Building on the current findings, future research could further investigate informant differences using item response theory approaches to examine whether children and parents systematically differ in their responses to similarly worded items, particularly in comparing category thresholds and differential item functioning [21, 31]. The complexity and partial inconsistency of the latent patterns across methods underline the need for replication studies. Beyond exploratory (data-driven) analyses, such studies could use the profiles found in the current study to formalize replication efforts by applying confirmatory latent class analysis [51] or by testing the similarity of latent profiles [52]. In particular, future research should examine the role of external variables such as age and the role of gender in the child-parent-dyads. These findings would certainly be interesting, even though previous research suggests that there were no significant differences in mother-adolescent and father-adolescent correlations [22] and fathers were found to be equally adept at recognizing their children’s problems/symptoms as the mothers [21]. It would also be valuable to examine how family characteristics shape discrepancies between informants, and what these discrepancies may reveal about their potential directionality.

## Supplementary Information

Below is the link to the electronic supplementary material.

Supplementary Material 1

Supplementary Material 2

## Data Availability

The data that supports the findings of this study are available from the Robert Koch Institute upon reasonable request.

## Footnotes

1 Following Liu et al. [23], PABAK is classified as: 0.01 to 0.2, slight agreement; 0.21 to 0.40, fair agreement; 0.41 to 0.60, moderate agreement; 0.61 to 0.80 substantial agreement; 0.81 to 0.99, almost perfect agreement. Note, that the results for PABAK in Liu et al. [23] are based on a special version for ordinal categories where weights have to be specified, and the values may not be completely comparable.
