# Supplementary material for: Patterns of agreement between children and parents in SDQ subscales: latent class and latent profile analyses
Source: Child Adolesc Psychiatry Ment Health. 2026 Nov 11;20:156. doi: 10.1186/s13034-026-01206-1 (PMC13769178; doi:10.1186/s13034-026-01206-1)
Supplement: Supplementary file 1 [file 13034_2026_1206_MOESM1_ESM.pdf]

Supplementary material for:

Keller/Langmeyer-Tornier/Reinecke/Schlack:

Patterns of agreement between children and parents in SDQ subscales: Latent class and latent profile analyses

Subscale: Peer Problems

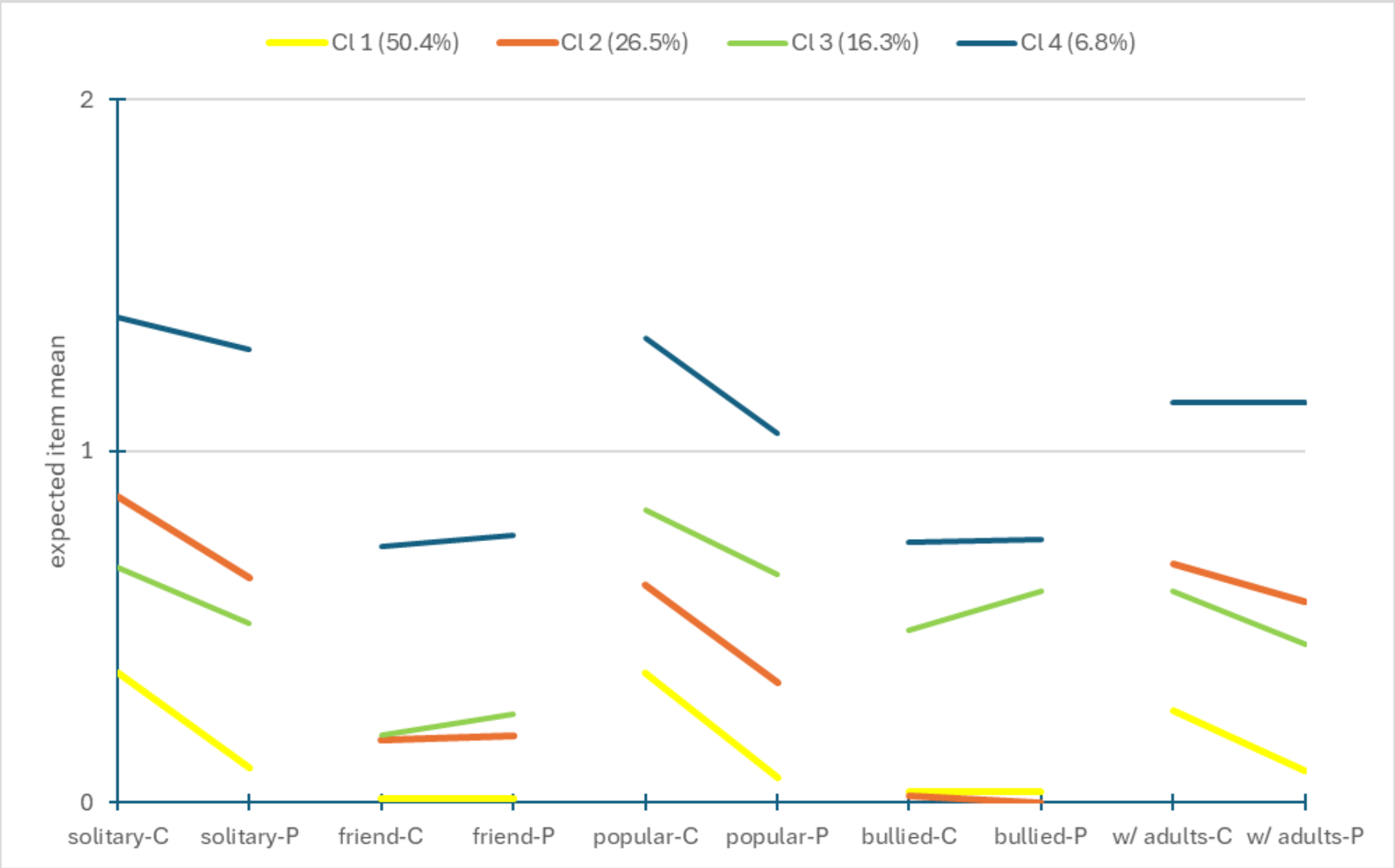

Figure S1: Expected item means for the 4-class solution for the subscale Peer Problems.

Note: Items are ordered pairwise, first rating of the child (-C), then rating of the parent (-P).

Subscale: Hyperactivity/Inattention

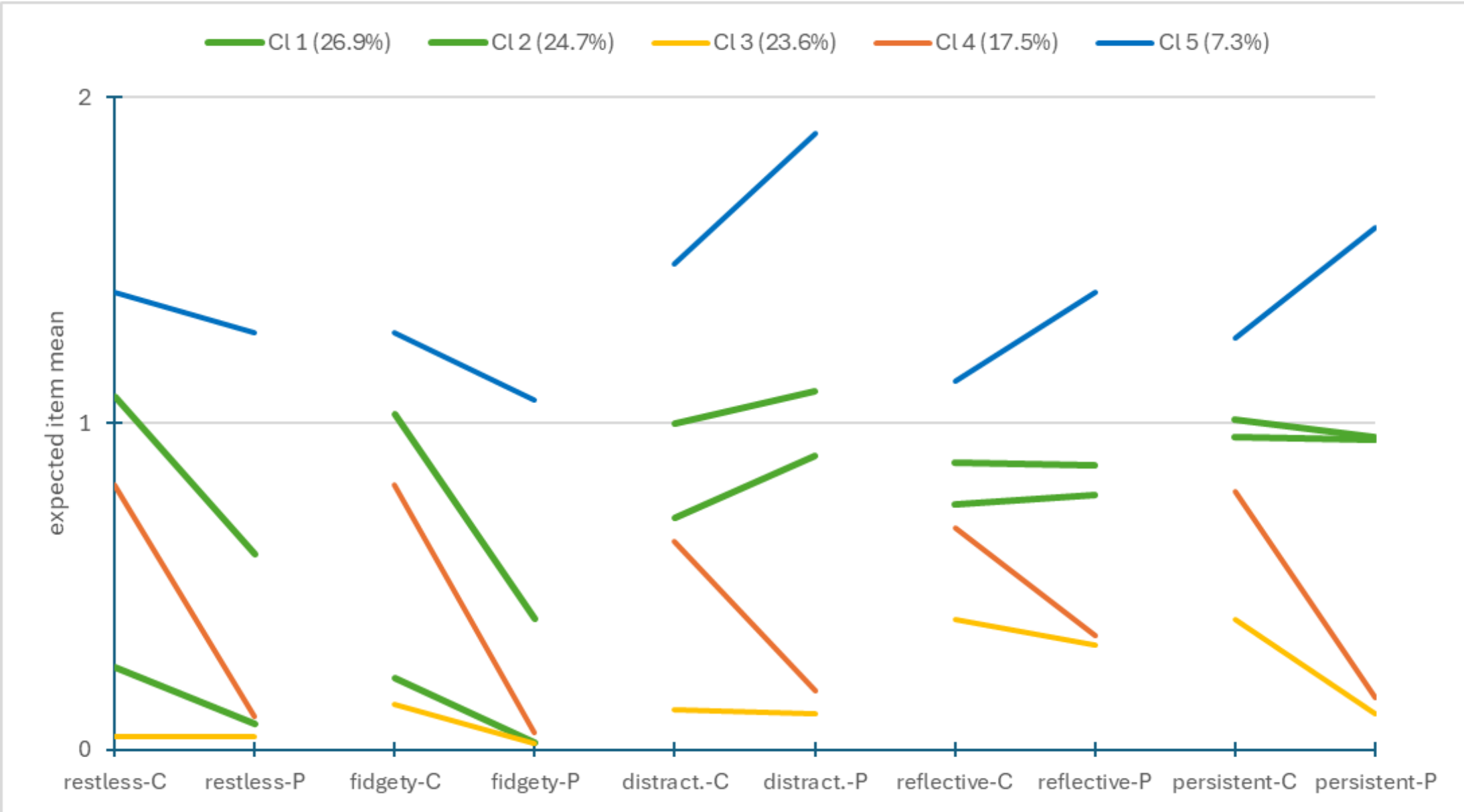

Figure S2: Expected item means for the 5-class solution for the subscale Hyperactivity/Inattention.
Note: Items are ordered pairwise, first rating of the child (-C), then rating of the parent (-P).

Subscale: Conduct Problems

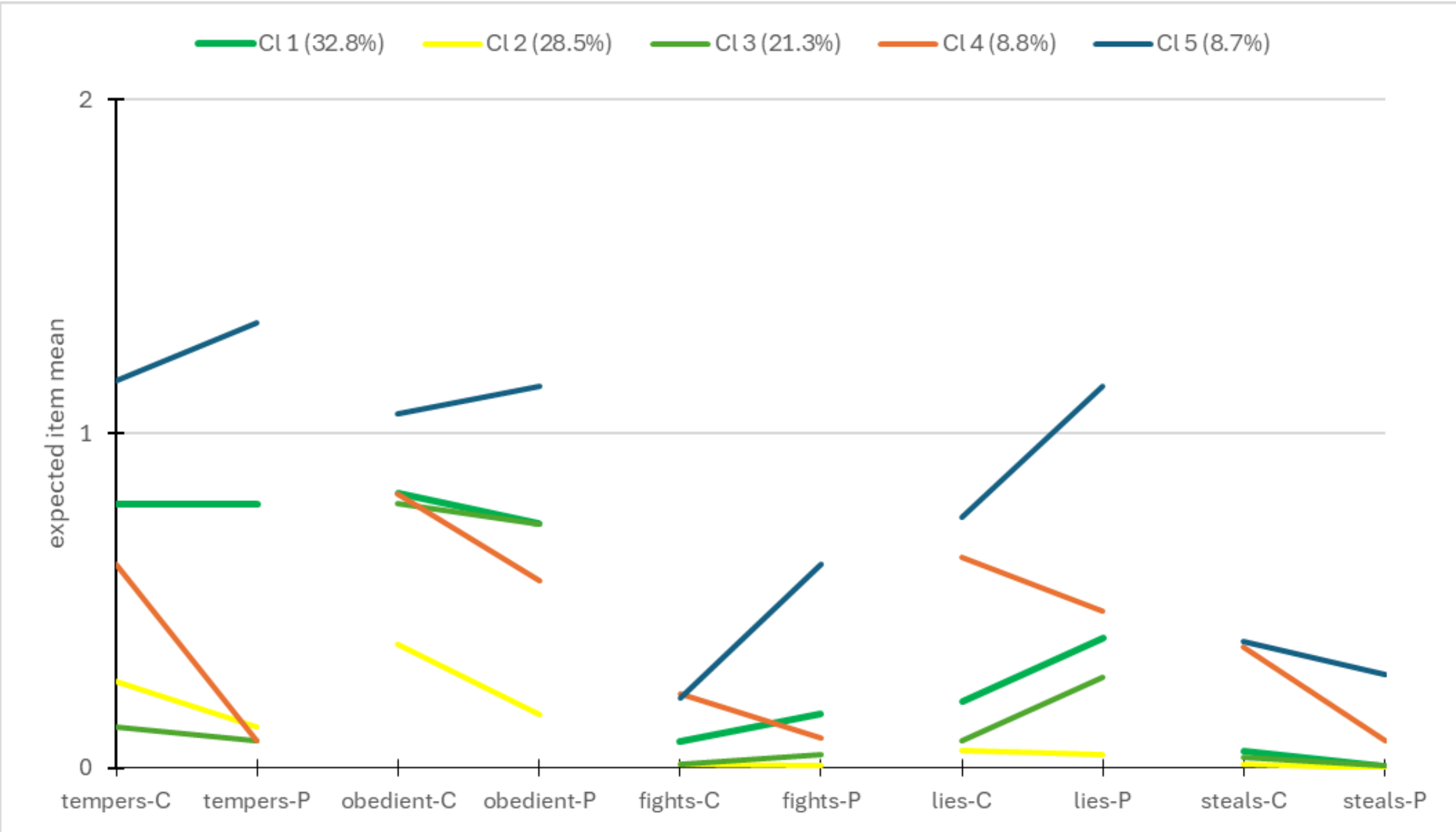

Figure S3: Expected item means for the 5-class solution for the subscale Conduct Problems.
Note: Items are ordered pairwise, first rating of the child (-C), then rating of the parent (-P).

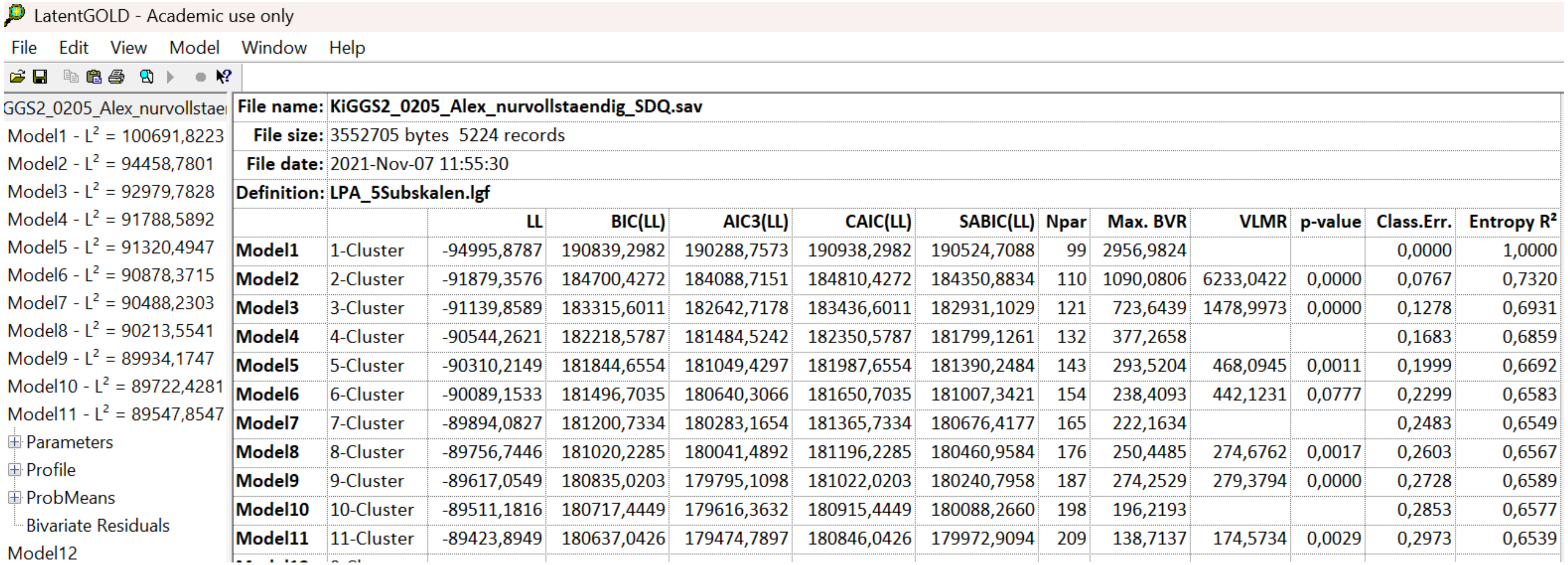

File name: KiGGS2_0205_Alex_nurvollstaendig_SDQ.sav
File size: 3552705 bytes 5224 records
File date: 2021-Nov-07 11:55:30
Definition: LPA_5Subskalen.lgf

		LL	BIC(LL)	AIC3(LL)	CAIC(LL)	SABIC(LL)	Npar	Max. BVR	VLMR	p-value	Class.Err.	Entropy R^2
**Model1**	1-Cluster	-94995,8787	190839,2982	190288,7573	190938,2982	190524,7088	99	2956,9824			0,0000	1,0000
**Model2**	2-Cluster	-91879,3576	184700,4272	184088,7151	184810,4272	184350,8834	110	1090,0806	6233,0422	0,0000	0,0767	0,7320
**Model3**	3-Cluster	-91139,8589	183315,6011	182642,7178	183436,6011	182931,1029	121	723,6439	1478,9973	0,0000	0,1278	0,6931
**Model4**	4-Cluster	-90544,2621	182218,5787	181484,5242	182350,5787	181799,1261	132	377,2658			0,1683	0,6859
**Model5**	5-Cluster	-90310,2149	181844,6554	181049,4297	181987,6554	181390,2484	143	293,5204	468,0945	0,0011	0,1999	0,6692
**Model6**	6-Cluster	-90089,1533	181496,7035	180640,3066	181650,7035	181007,3421	154	238,4093	442,1231	0,0777	0,2299	0,6583
**Model7**	7-Cluster	-89894,0827	181200,7334	180283,1654	181365,7334	180676,4177	165	222,1634			0,2483	0,6549
**Model8**	8-Cluster	-89756,7446	181020,2285	180041,4892	181196,2285	180460,9584	176	250,4485	274,6762	0,0017	0,2603	0,6567
**Model9**	9-Cluster	-89617,0549	180835,0203	179795,1098	181022,0203	180240,7958	187	274,2529	279,3794	0,0000	0,2728	0,6589
**Model10**	10-Cluster	-89511,1816	180717,4449	179616,3632	180915,4449	180088,2660	198	196,2193			0,2853	0,6577
**Model11**	11-Cluster	-89423,8949	180637,0426	179474,7897	180846,0426	179972,9094	209	138,7137	174,5734	0,0029	0,2973	0,6539

Figure S4: Screen shot of the fit criteria for the 1- to 11-class (cluster) solutions of the Latent Profile Analysis with five SDQ subscales.

Table S1: Differences in SDQ subscales between child and parent ratings, separated for the six latent profiles (t-test for dependent data).

Class modal		Emotional Problems (child)	Emotional Problems (parent)	Peer Problems (child)	Peer Problems (parent)	Hyperact./ Inattention (child)	Hyperact./ Inattention (parent)	Conduct Problems (child)	Conduct Problems (parent)	Prosocial Behavior (child)	Prosocial Behavior (parent)
1	Mean	2,11	1,12	1,05	,35	3,98	2,79	1,41	1,22	8,51	8,51
	Diff. Mean / ES	0,99	,53	0,70	,63	1,19	,57	0,19	,14	0,01	,01
2	Mean	2,54	1,97	2,03	1,74	4,96	4,38	2,49	2,89	7,16	6,71
	Diff. Mean / ES	0,56	,25	0,29	,17	0,57	,25	-0,39	-,23	0,44	,22
3	Mean	1,88	,80	1,19	,58	1,46	,69	,56	,37	9,30	9,40
	Diff. Mean / ES	1,08	,65	0,61	,50	0,77	,50	0,19	,23	-0,10	-,09
4	Mean	5,20	3,33	3,51	2,40	3,27	1,89	1,70	1,18	8,37	8,52
	Diff. Mean / ES	1,87	,70	1,11	,55	1,38	,71	0,52	,35	-0,15	-,09
5	Mean	1,46	,65	2,24	1,60	2,04	1,26	1,29	1,15	7,11	7,27
	Diff. Mean / ES	0,81	,53	0,65	,40	0,78	,45	0,14	,10	-0,16	-,07
6	Mean	4,68	5,04	4,41	4,55	5,72	6,04	3,65	4,20	6,87	6,02
	Diff. Mean / ES	-0,36	-,13	-0,14	-,06	-0,32	-,13	-0,56	-,23	0,84	,36
Total	Mean	2,66	1,70	1,98	1,36	3,40	2,52	1,60	1,52	8,11	8,04
	Diff. Mean / ES	0,96	,45	0,61	,39	0,88	,43	0,08	,05	0,07	,04

Note: class 1: n = 1417; class 2: n = 1009; class 3: n = 1034; class 4: n = 774; class 5: n = 708; class 6: n = 282; Total: n = 5224; ES = effect size (red: ES > .40; green: ES < .20)

Pairwise cross-tabulations between latent classes

Table S2: Association between each pair of the latent class solutions in the five SDQ subscales.

Subscales	Emotional	Peer	Hyperact.	Conduct	Prosocial
Emotional Problems	-	688.7	314.2	398.6	66.4
Peer Problems	.210	-	294.8	424.8	258.1
Hyperactivity/Inattention	.123	.137	-	1228.0	424.3
Conduct Problems	.138	.165	.242	-	788.1
Prosocial Behavior	.065	.128	.165	.224	-

Note: upper triangle: chi²-values (all p < .001, df = various, 9 – 16); lower triangle: Cramer's *V*.

Table S3: Differences in sex and age for the LPA/LCA classes.

Model/SDQ Subscale	%female	Age mean (SD)	Short Description of Classes
**Latent Profile Model**	50.5%	13.59 (3.75)	1: Low internalizing/medium hyperactivity – C > P
	38.7%	13.49 (3.61)	2: Elevated externalizing with agreement
	64.4%	13.93 (4.00)	3: Low symptoms with slight disagreement
	72.6%	14.31 (3.64)	4: High internalizing and externalizing, all C > P
	35.9%	14.31 (3.67)	5: Slightly elevated with moderate disagreement
	48.9%	13.62 (3.94)	6: High symptom burden with agreement
	chi²=343.61	F=29.96	
	V=.256	eta²=.028	
**LCA Model (item-level)**			
Emotional Problems	41.0%	13.81 (3.83)	1: low-symptomatic – concordant
	70.5%	14.35 (3.74)	2: Child-reported distress – C > P
	55.8%	13.73 (3.90)	3: high somatic-focused symptoms – concordant
	47.3%	13.42 (3.65)	4: Parent-reported concerns – P > C
	78.3%	14.03 (3.77)	5: high symptomatic – concordant
	chi²=381.57	F=24.42	
	V = .27	eta²=.018	
Peer Problems	53.9%	13.71 (3.90)	1: Low peer problems – concordant
	50.9%	14.32 (3.52)	2: Child-reported mild peer difficulties – C > P
	50.2%	13.36 (3.54)	3: Moderate peer issues – concordant
	47.3%	13.92 (3.99)	4: high parent-reported peer problems – P > C
	chi²=8.38,	F=47.20	
	p=.039,	eta²=.026	
	V=.040		
Hyperactivity/Inattention	49.7%	13.96 (3.72)	1: Inattentive without hyperactivity – concordant
	43.6%	13.53 (3.69)	2: Inattentive and hyperactive – concordant
	61.3%	14.13 (3.83)	3: Low symptoms – slightly C > P
	61.4%	14.06 (4.10)	4: More symptoms reported by child – C > P
	38.8%	13.02 (3.10)	5: Highly hyperactive – concordant (7.3%)
	chi²=141.17;	F=36.12	
	V=.16	eta²=.027	

Model/SDQ Subscale	%female	Age mean (SD)	Short Description of Classes
Conduct Problems	53.1%	13.86 (3.94)	1: Irritable and oppositional – concordant
	56.7%	13.85 (3.92)	2: Low conduct problems – concordant
	51.5%	14.01 (3.80)	3: Disobedient only – concordant
	38.8%	13.64 (3.76)	4: Child-reported conduct issues – C > P
	43.0%	13.52 (3.34)	5: Severe conduct problems – slightly P > C
	chi²=53.10;	F=5.47; p=.0002	
	V=.10	eta²=.004	
Prosocial Behavior	63.7%	13.82 (3.99)	1: High prosocial – concordant
	50.9%	13.86 (3.85)	2: Child-reported medium prosocial – C > P
	42.5%	13.88 (3.80)	3: Parent-reported medium prosocial – P > C
	35.2%	13.85 (3.58)	4: Low prosocial – concordant
	chi²=226.40	F=0.24; p=.866	
	V=.208	eta²=.000	
**LCA 2nd order**	57.4%	14.01 (3.97)	1: Well-Adjusted Concordant Group
	42.1%	13.71 (3.71)	2: Mild Externalizing Concordant Group
	50.1%	13.75 3.85)	3: Pronounced Externalizing Concordant Group
	70.2%	14.23 (3.76)	4: Heterog. Cross-Domain Concord. Difficulty Group
	43.8%	13.32 (3.40)	5: Parent-Reported Difficulty (P > C) Group
	54.8%	14.00 (3.89)	6: Child-Reported Difficulty (C > P) Group
	chi²=160.79	F=15.94	
	V=.175	eta²=.015	

Note: All statistical tests are significant with p < .0001 except where p-values are provided; conventional cutoffs for effect size Cramer's V: 0.10 = small; 0.30 = medium; 0.50 = high; for effect size eta²: 0.01 = small; 0.06 = medium; 0.14 = high.
